# Guided Electrokinetic Assembly of Functionalized Latex Beads for Fluorescence Signal Enhancement Using AC Electro-Osmosis

**DOI:** 10.3390/s26165264

**Published:** 2026-08-20

**Authors:** Tuo Zhou, Alfonso Shin, Lawrence Kulinsky

**Affiliations:** 1 Materials and Manufacturing Technology, University of California, Irvine, CA 92697, USA; tuoz3@uci.edu; 2 Department of Biomedical Engineering, University of California, Irvine, CA 92697, USA; alfons1@uci.edu; 3 Department of Mechanical and Aerospace Engineering, University of California, Irvine, CA 92697, USA

**Keywords:** biomedical diagnostic, enzyme-linked immunosorbent assay, electrokinetic assembly

## Abstract

Fluorescence-based immunoassays are widely used for sensitive and specific biomarker detection; however, further improvements in sensitivity remain desirable for detecting low-abundance analytes without increasing assay complexity. In this work, we present a proof-of-concept demonstration of guided electrokinetic assembly of functionalized latex beads as a post-assay signal enhancement strategy using alternating-current electro-osmosis (ACEO). Carboxyl-modified 1 μm polystyrene beads conjugated with Alexa Fluor 647-labeled anti-IgG were localized within lithographically defined windows on carbon interdigitated electrode arrays, producing localized fluorescence enhancement through physical bead localization without enzymatic amplification or additional labeling chemistries. Compatibility of the approach with fluorescence-based immunoassays was demonstrated through adaptation of a TNF-α ELISA workflow. Electro-osmotic localization of functionalized bead conjugates was achieved within 120 s, producing an approximately 12-fold increase in corrected total fluorescence relative to the corrected signal of the pre-electro-osmosis condition while demonstrating negligible enrichment of unbound fluorescent protein. Application of the platform to a TNF-α sandwich immunoassay yielded an approximately 5.5-fold enhancement in fluorescence signal, and robust bead localization was maintained across anti-IgG concentrations ranging from 1 to 4 μg/mL. These findings demonstrate that guided electrokinetic bead localization provides an effective signal enhancement strategy for fluorescence-based immunoassays and represents a promising approach for improving the detection of low-abundance analytes.

## 1. Introduction

Immunoassays have long served as a cornerstone of clinical diagnostics and biomedical research due to their ability to sensitively and specifically detect target analytes in complex biological samples [1,2]. Techniques such as enzyme-linked immunosorbent assays (ELISAs) and fluorescent immunoassays (FIAs) commonly rely on immobilization of capture antibodies onto planar solid supports to facilitate binding of analytes of interest. While these approaches are widely used, assay performance is often limited by mass transport of analyte molecules from the bulk solution to the immobilized surface [3]. As analytes are depleted near the reaction interface, diffusion becomes the dominant transport mechanism, slowing reaction kinetics and ultimately limiting assay sensitivity.

Mass transport-based strategies for enhancing assay sensitivity can be broadly classified into two categories: pre-concentration and post-assay enhancements. Pre-concentration refers to the enrichment or localization of target analytes, or analyte-containing complexes, from a bulk sample into a smaller effective detection volume prior to measurement, thereby increasing the likelihood of probe-target interactions and improving detection sensitivity [4]. This enrichment may be achieved either by concentrating analytes directly or by employing high-surface-area functionalized particles that increase target capture efficiency. In contrast, post-assay enhancements refer to amplification of the detection signal after the biological recognition event has been completed. Rather than increasing the amount of captured analyte, post-assay enhancement increases the local density of signal-generating species, such as fluorescently labeled beads, within a confined detection region to enhance the measured signal [5].

Bead-based assays are particularly well suited to both strategies due to their large surface-area-to-volume ratio, promoting efficient target capture while simultaneously providing mobile signal-generating carriers that can be manipulated before or after analyte recognition. Functionalized micro- and nanobeads therefore provide versatile platforms for pre-concentration, post-assay enhancement, or both.

Magnetic nanoparticles (MNPs), such as paramagnetic Dynabeads™, are widely used for high-sensitivity diagnostic applications because they can be manipulated by an external magnetic field to enrich target-bound complexes [6,7,8,9,10,11,12]. In a typical MNP-based pre-concentration workflow, target-bound beads are magnetically collected and resuspended into a smaller volume prior to detection, enabling enrichment of analyte-containing complexes while removing unbound reagents [13,14,15,16,17]. Similarly, multiplexed bead technologies such as Luminex^®^ enable simultaneous detection of hundreds of analytes within a single sample [18,19]. Despite their effectiveness, many bead-based platforms rely on magnetic manipulation, proprietary instrumentation, or specialized detection systems that increase assay complexity and cost [20,21].

Compared with conventional magnetic bead-based immunoassays, which require magnetic collection, repeated resuspension, and transfer of target-bound beads prior to detection, electrokinetic manipulation provides an alternative strategy for bead handling and concentration. Pre-concentration through the electrokinetic manipulation of particles has been well noted, with advantages such as shorter detection time, increased localized concentrations, and improved sensitivity [22]. Electrically driven preconcentration strategies such as field-amplified sample stacking (FASS) have similarly been investigated to increase localized analyte concentrations in microfluidic systems. In FASS, differences in buffer conductivity generate local electric-field gradients that alter the migration velocity of charged analytes, resulting in their accumulation at the interface between regions of differing conductivity [23]. Previous studies have demonstrated that the combination of dielectrophoresis (DEP) and alternating-current electro-osmosis (ACEO) can direct suspended microparticles into predefined regions on interdigitated electrode arrays [24,25]. In a nonuniform AC electric field, dielectrophoretic forces act on polarized particles according to their electrical properties relative to the suspending medium, while electro-osmotic vortices transport particles toward patterned electrode windows. Together, these forces driving flow manipulation selectively localize and retain bead-bound complexes within microscale detection regions while excluding freely diffusing proteins and fluorescent species [24,25,26,27]. Because this enrichment occurs directly within the original sample volume, electrokinetic manipulation provides post-assay enhancement by the localization of bead-bound complexes and may offer opportunities for future simplification of bead-based assay workflows.

In addition to post-assay enhancement via bead concentration and localization, the microscale vortices generated by electro-osmotic flow enhance convective mass transport and accelerate binding kinetics. Continuous recirculation of suspended latex beads repeatedly exposes bead-bound probes to fresh analyte from non-depleted regions, increasing the probability of target capture while reducing diffusion limitations. Because electro-osmotic flow is electrically controlled, this mixing strategy may also facilitate intermediate binding steps while minimizing analyte loss associated with centrifugation- or magnet-based processing. Consequently, electrokinetic manipulation has the potential to simplify bead-based immunoassay workflows while improving reaction efficiency, analyte recovery, and signal-to-noise ratio. In this work we focus on post-assay enhancement via electrokinetic assembly of the beads as opposed to quantified enhancement of binding kinetics or capture of the target analyte [4]. In prior work [25], we identified that dielectrophoresis works well for the guided microassembly of beads and biological cells with diameters of several microns or larger, while electro-osmosis can be utilized for effective assembly of polymer particles with diameters of 1 micron and below. Therefore, since we employed one-micron beads in this study, we utilized electro-osmosis. Experimental verification was undertaken to confirm that dielectrophoresis did not work for the assembly of one-micron beads in the wide range of frequencies, while electro-osmosis for the applied potential of 2 V and applied frequency of 200 Hz worked well to assemble the beads in the diluted phosphate-buffered saline (PBS). If larger functionalized beads are used, employment of dielectrophoresis instead of (or in addition to) electro-osmosis should be considered.

Electrokinetic post-assay enhancement via electrokinetic assembly of the beads represents a complementary strategy for improving assay sensitivity following completion of the biological recognition event. Post-assay bead concentration enhances the measured signal by physically localizing signal-generating species, such as fluorescently labeled microbeads, within a confined detection region. This principle has been extensively applied to increase the local density of fluorophores and improve signal intensity, demonstrating 100-fold improvements in sensitivity relative to conventional ELISA readers [28,29]. Electrokinetic forces enable this localization through controlled transport and confinement of suspended particles into pre-defined microscale regions, allowing signal-generating species to be concentrated without additional processing steps or changes to sample composition. Together, these strategies improve sensitivity through both increased target capture efficiency and physical amplification of the measured fluorescence signal.

In this work, we investigate guided electrokinetic assembly primarily as a strategy for post-assay fluorescence signal enhancement through the concentration and localization of bead-bound complexes within predefined detection regions. Although electrokinetic flow may also enhance convective mass transport during binding, the present experiments are not designed to independently quantify the contribution of enhanced mass transport to assay signal. These complementary mechanisms are implemented on a single microfluidic platform that integrates bead manipulation, target concentration, and fluorescence detection without intermediate magnetic separation or bead transfer. Carboxyl-modified latex beads conjugated with Alexa Fluor 647-labeled anti-IgG were localized within patterned electrode windows using flow manipulation of combined electro-osmotic and dielectrophoretic forces. The specificity and signal enhancement capabilities of the technique were evaluated using fluorescent and non-fluorescent controls, followed by application to a TNF-α sandwich immunoassay. Fluorescence intensity was quantified using ImageJ (version 1.54p) analysis to evaluate the signal enhancement achieved via electrokinetic post-assay concentration and localization of functionalized microbeads.

## 2. Materials and Methods

### 2.1. Fabrication of the Electrode Arrays

**Figure 1:**
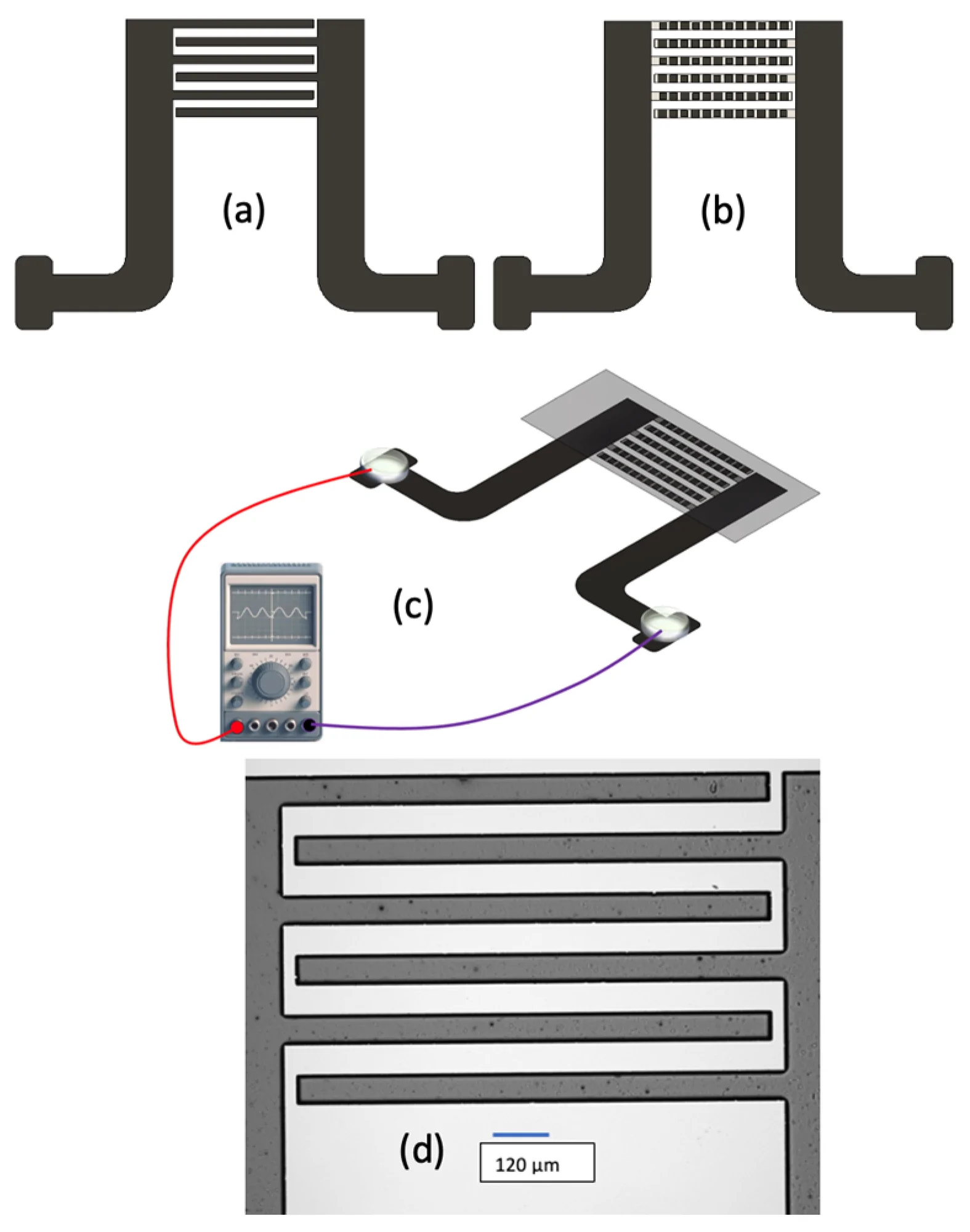
(**a**) The sketch of the carbon IDEA; (**b**) sketch of IDEA coated with a thin layer of photoresist with patterned windows exposing electrode underneath; (**c**) schematics of experimental setup including the IDEA chip (containing the analyte solution filled with functionalized microbeads) connected to the function generator; (**d**) the optical microscopic picture of the glassy carbon IDEA with six interdigitated electrodes.

Glassy carbon inter-digitated electrode arrays (IDEAs) were fabricated by conventional photolithography process followed by pyrolysis. Four-inch diameter silicon wafers covered by a thin layer of thermal oxide were used as the substrate in this study (University Wafer, South Boston, MA, USA). SU-8 2025 photoresist (MicroChem Corp., Newton, MA, USA) was spin-coated onto the silicon wafers using a Laurell photoresist spinner (Laurell Technologies Corp., North Wales, PA, USA) at an angular velocity of 500 rpm for 10 s followed by 4000 rpm spin for 30 s. After the spin coating with photoresist the wafers were soft-baked at 95 °C for 5 min on a hot plate (Dataplate, Inc., Algona, IA, USA). Next, the SU-8 resist was exposed through a plastic mask (CAD/Art Services, Inc., Bandon, OR, USA) via a UV flood exposure system (Newport Corp., Irvine, CA, USA) for 6 s at an energy intensity of 10 mW/cm^2^. The wafers were then post-baked at 95 °C for 5 min followed by immersion in SU-8 developer (MicroChem Corp., Newton, MA, USA) for 3 min followed by hard baking at 95 °C for 45 min on the hot plate. After the fabrication, the SU-8 patterns were carbonized in a furnace (Thermo Fisher Scientific, Waltham, MA, USA) with an optimal thermal profile of 25 °C for 2 h, then 300 °C for 1 h, followed by 900 °C for 1 h, and cooling down to 25 °C, with a ramp rate of 10 °C/h. The height and width of the electrode fingers were measured using a Dektak 3 profilometer (Veeco Instruments Inc., Plainview, NY, USA). Figure 1 presents the sketch of the interdigitated electrode array where the width of each electrode and the distances between each adjacent electrode are both 120 µm, and the height of the electrode is 4 µm.

**Figure 2:**
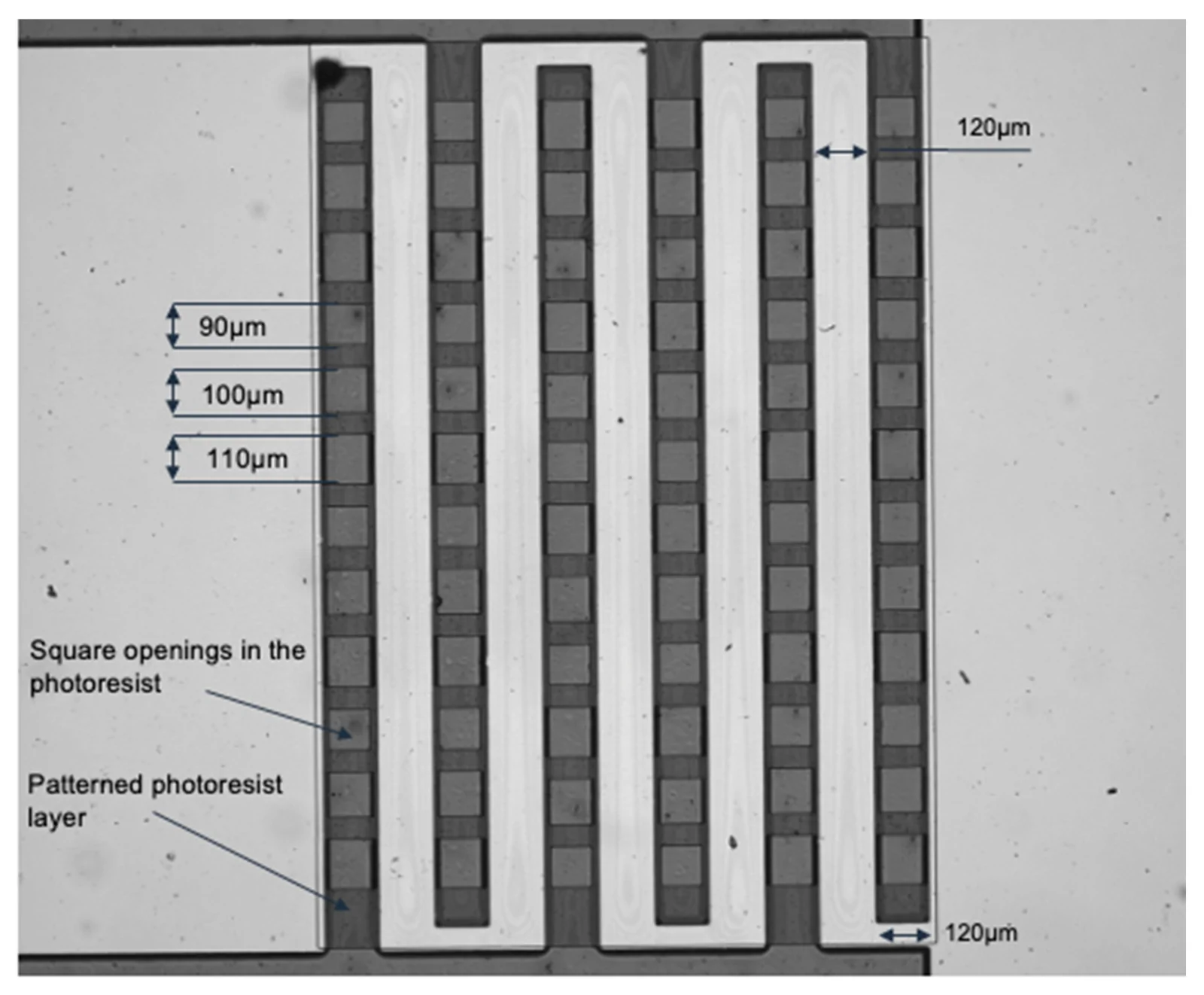
The array of windows patterned on the top of electrode fingers.

After the interdigitated electrode array was fabricated as described above, a layer of SU-8 2002 photoresist was spin-coated to cover the glassy carbon IDEA at an initial speed of 500 rpm for 10 s followed by the spin of 1000 rpm for 30 s. The wafer was soft-baked at 95 °C for 3 min and then exposed through an iron oxide mask (Front Range Photomask, Las Vegas, NV, USA) for 4 s at an energy intensity of 10 mW/cm^2^ using the MA5 mask aligner (Karl Süss, Garching, Germany), followed by the post baking at 95 °C for 3 min. The wafers were submerged in the SU-8 developer for 5 min, followed by the hard baking at 120 °C for 30 min. An array of square windows with sides of 90 μm, 100 μm, and 110 μm opened in the photoresist on top of electrode fingers was thus produced (see Figure 2). While slightly different dimensions of the windows were selected to optimize the electrokinetic assembly, there was no appreciable difference in bead gathering effect between different windows.

### 2.2. Anti-IgG—Alexafluor-647 Bead Preparation

Carboxyl-modified latex (CML) polystyrene beads (1 μm; Thermo Fisher Scientific, Waltham, MA, USA) were prepared at 2.5 mL (40 mg/mL) and diluted with 10 mL 0.025 M 2-(N-morpholino) ethanesulfonic acid (MES) buffer (Sigma-Aldrich, St. Louis, MO, USA). Samples were then centrifuged at 3000 rpm for 20 min (mySPIN 12, Thermo Fisher Scientific, Waltham, MA, USA). Supernatant was then removed from the particles and resuspended in 5 mL 0.025 M MES buffer at an approximate concentration of 20 mg/mL.

Bead controls were prepared according to the passive adsorption protocol listed by Thermo Fisher Scientific (see Supplementary Materials) [30] to produce a positive control consisting of conjugation of Goat anti-Human IgG Fc Recombinant Secondary Antibody, Alexafluor-647 (Thermo Fisher Scientific, Waltham, MA, USA) and a negative control consisting of conjugation of Goat anti-Human IgG Fc Cross-Adsorbed Secondary Antibody, HRP (Thermo Fisher Scientific, Waltham, MA, USA) on the aforementioned 1 μm latex beads. Anti-human IgG antibody controls were prepared at a concentration of 4 μg/mL in 0.025 M MES buffer and at a 1:1 dilution with 500 μL of prepared latex beads. Samples were then incubated at 25 °C overnight for 10 h and centrifuged at 3000 rpm for 20 min. Supernatant was then removed and resuspended with 1 mL 1× PBS (Thermo Fisher Scientific, Waltham, MA, USA) and centrifuged again to sediment the particles. This wash step was repeated for a total of three washes. Bead samples were then blocked in 250 μL 1× StartingBlock™ (PBS) blocking buffer (Thermo Fisher Scientific, Waltham, MA, USA) for 2 h at 25 °C. Samples were then centrifuged again to sediment the particles at 3000 rpm for 20 min and washed in 400 μL 1× PBS. Washes were performed a total of three times, and beads were resuspended in 1 mL 1× PBS. Prior to electro-osmosis testing on the electrodes, bead samples were immediately prepared at a 1:10 dilution in UltraPure™ DNase/RNase-Free Distilled Water (Thermo Fisher Scientific, Waltham, MA, USA).

### 2.3. Experimental Setup

After the fabrication of the carbon electrode and layer of windows, wires were soldered to the carbon contact pads of the IDEA chips using indium. The double-sided adhesive tape (approximately 100 microns thick) (3M, St. Paul, MN, USA) was used to create an enclosure for liquid sample atop of the electrode array. Then, 10 μL of the 1:10 bead dilution was deposited on top of the electrodes inside the enclosure. A glass cover slide was placed over the chip to facilitate microscopic observation and reduce sample evaporation. A microscopic observation was conducted using a Nikon Eclipse microscope (Nikon Corporation, Tokyo, Japan) and recorded using SPOT Basic image capture software (version 5.2; SPOT Imaging, Sterling Heights, MI, USA). IDEA chips were connected to a function generator (Stanford Research System, Sunnyvale, CA, USA) to produce AC field at a voltage of 2 V peak-to-peak and frequency of 200 Hz. The increase in voltage and decrease in frequency leads to a more vigorous electro-osmotic eddies, and the applied voltage and frequencies were selected experimentally to ensure collection of the microbeads within the windows while minimizing intensity of electro-osmotic eddies in order to avoid shear stresses on the beads.

## 3. Results and Discussion

### 3.1. Validation of the Electro-Osmotic Bead Concentration Technique for Use with Fluorescent Immunoassays

The physics of electrokinetic assembly of microbeads, including dielectrophoresis and electro-osmosis, and the selection of ACEO-driven flow as the most appropriate non-contact force for collection of 1-micron polystyrene beads were discussed previously [21].

**Figure 3:**
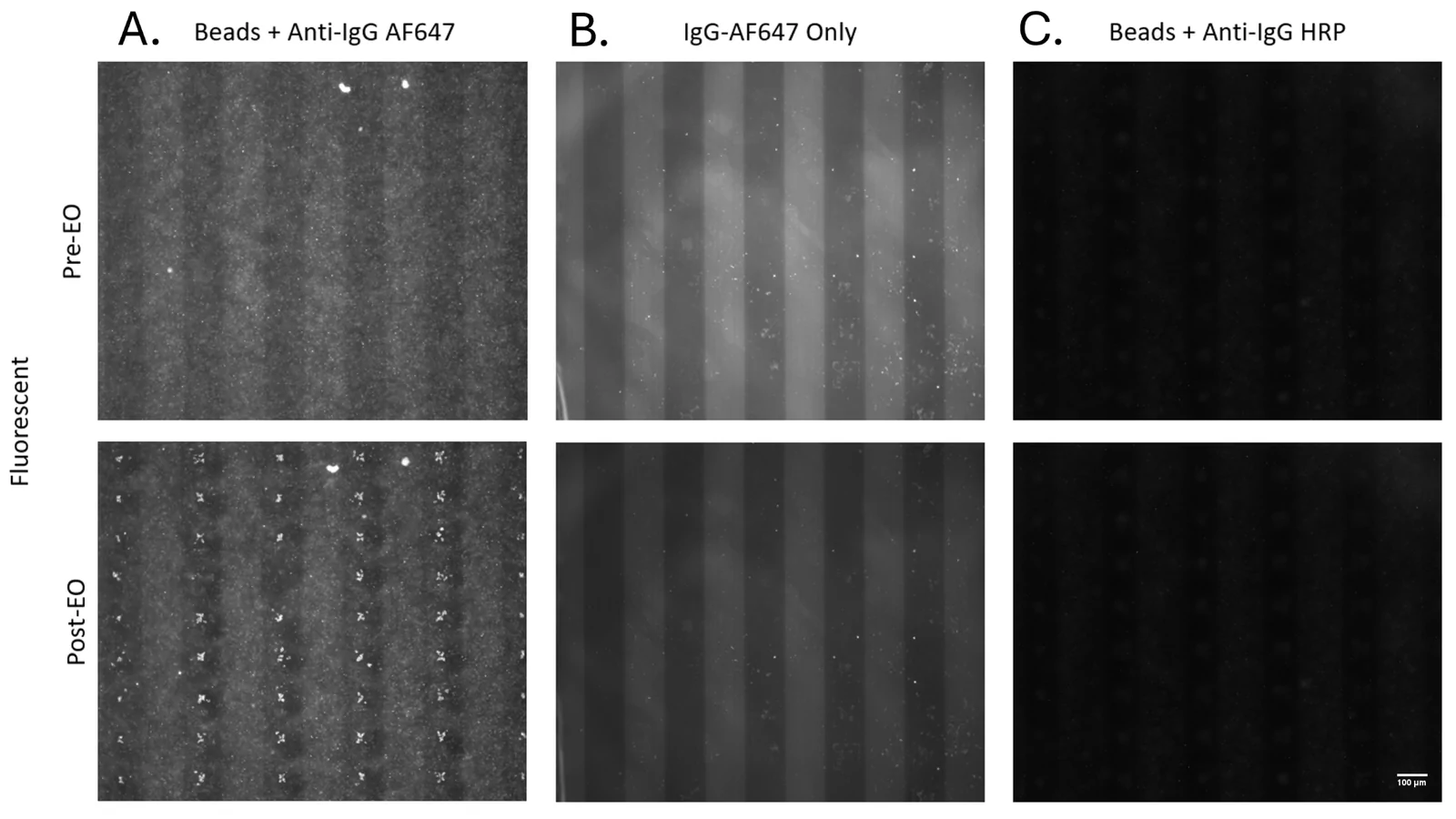
Representative fluorescence microscopy images of the positive and negative control conditions before (Pre-EO) and after (Post-EO) electro-osmosis. The positive control (**A**) exhibited a marked increase in fluorescence following electro-osmosis, consistent with successful concentration of fluorescently labeled beads. The negative controls included anti-IgG Alexa Fluor 647 without beads (**B**) and beads labeled with anti-IgG horseradish peroxidase (HRP) (**C**). Both negative controls exhibited minimal change in fluorescence with the windows before and after electro-osmosis, confirming that the observed fluorescence signal was specific to AF647-labeled beads rather than nonspecific background or device autofluorescence.

In the present study, the feasibility of the application of electro-osmotic bead concentration technique was first evaluated by three sets of controls in order to determine the biocompatibility of the electro-osmotic bead concentration technique with antibody-based detection techniques. Electro-osmotic experiments were conducted using a positive control (0.4 μg/mL Anti-IgG Alexafluor-647 conjugated beads) and negative control (0.4 μg/mL Anti-IgG Alexafluor-647 protein) of fluorescently conjugated protein to determine whether voltage and frequency settings were optimized for the solely specific capture of bead products in the electrode windows and microscope negative control (0.4 μg/mL Anti-IgG HRP conjugated beads) to determine that bead and protein reagents used in this experiment did not exhibit autofluorescence. Figure 3 demonstrates pre-electro-osmosis (Pre-EO) images of 9 × 5 array prior to application of electro-osmosis (at the 0 s time point) and post-electro-osmosis (Post-EO) images captured following application of electro-osmosis at the 120 s time point. ImageJ analysis was used to calculate the signal within the windows by subtracting the background signal outside the windows from the fluorescence within each window, then averaging the total fluorescence.

**Figure 4:**
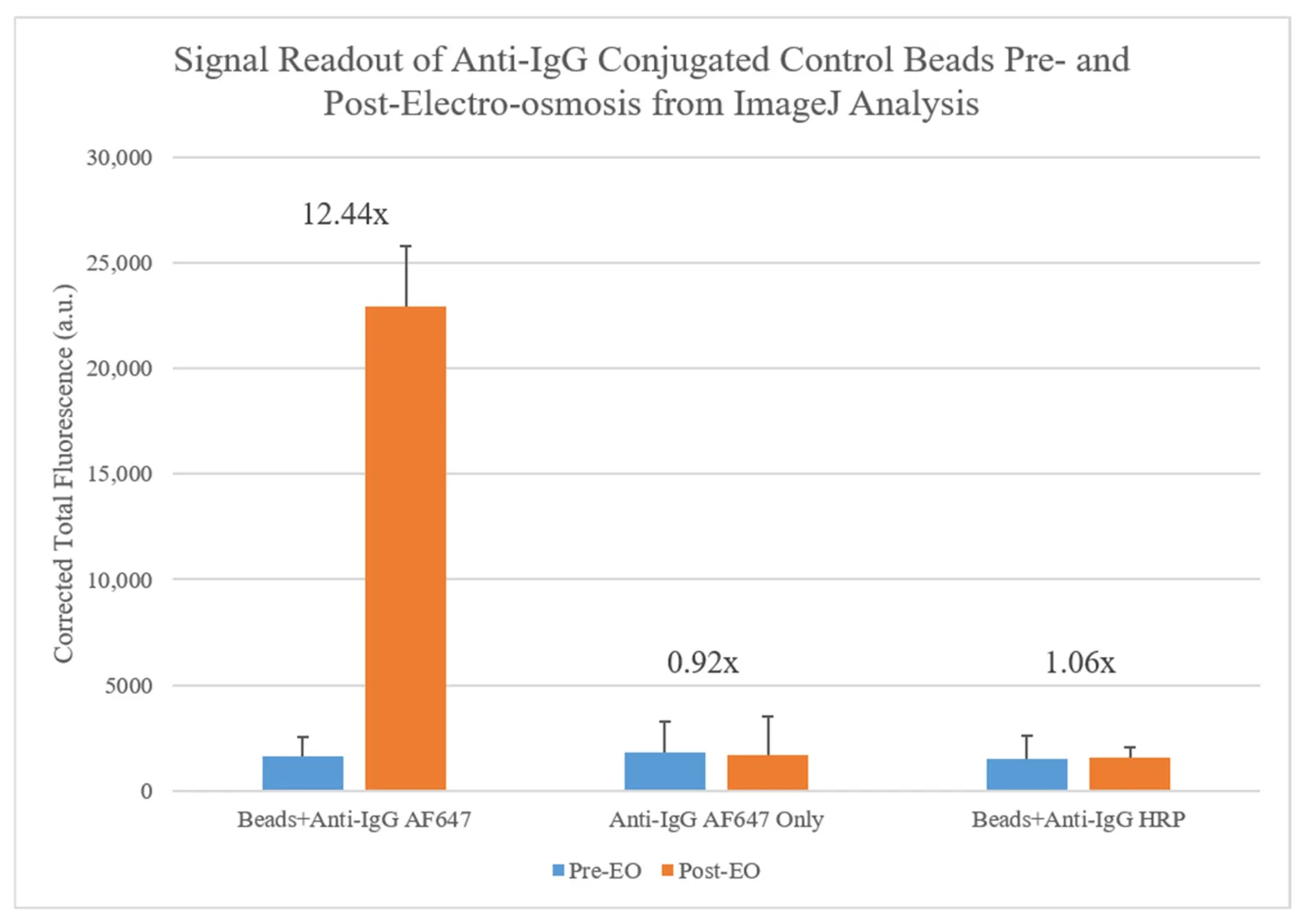
Validation of electro-osmotic concentration specificity using fluorescent and non-fluorescent controls. Corrected total fluorescence was measured before (Pre-EO) and after (Post-EO) electro-osmosis for fluorescent anti-IgG Alexa Fluor 647-conjugated beads (positive control), free anti-IgG Alexa Fluor 647 protein without beads, and anti-IgG HRP-conjugated beads (fluorescence-negative control) with fluorescence enrichment factors of 12.44, 0.92, and 1.06 respectively. The fluorescence enrichment factor was calculated as Post-EO CTCF/Pre-EO CTCF. Data are presented as the mean ± standard deviation (SD) from three repeated measurements of the same window (*n* = 3). Descriptive statistical analyses were performed using Microsoft Excel (Microsoft Corporation, Redmond, WA, USA).

To evaluate the specificity and biocompatibility of electro-osmotic bead concentration for protein-based assays, three control conditions were examined: (1) latex beads conjugated with anti-IgG Alexa Fluor 647 (positive control), (2) free anti-IgG Alexa Fluor 647 protein without beads, and (3) latex beads conjugated with anti-IgG HRP (fluorescence-negative control). Figure 4 presents the results of the corrected total fluorescence for Pre-EO and Post-EO three types of analytes, with corrected total fluorescence (CTCF) defined as the integrated fluorescence intensity of the localized bead region minus the product of the region area and the mean background fluorescence intensity. Fluorescence intensity measurements were quantified using ImageJ (National Institutes of Health, Bethesda, MD, USA) with calculations performed using CTCF = Integrated Density − (Area × Mean Background). Data are presented as the mean ± standard deviation (SD) from three repeated measurements of the same window (*n* = 3). Descriptive statistical analyses were performed using Microsoft Excel (Microsoft Corporation, Redmond, WA, USA).

The results confirmed that electro-osmosis is compatible with the detection of antibody-based protein and results in significant localization of beads within the windows of the electrode and produces an enhanced fluorescent readout. Following the application of electro-osmosis, a 12.44-fold increase in corrected total fluorescence was observed only in the positive control containing fluorescently labeled beads, with the fluorescence enrichment factor defined as the CTCF post-electro-osmosis relative to the CTCF pre-electro-osmosis. In contrast, free anti-IgG Alexa Fluor 647 protein exhibited minimal signal accumulation within the electrode windows and a Post-EO/Pre-EO CTCF ratio of approximately 0.92, indicating that electro-osmosis does not promote nonspecific concentration of unbound fluorescent protein that could generate false-positive signal. Additionally, the anti-IgG HRP-conjugated bead control demonstrated negligible fluorescence before and after electro-osmosis supported by a Post-EO/Pre-EO CTCF ratio of approximately 1.06, confirming the absence of significant bead autofluorescence. These findings demonstrate that signal enhancement is dependent on the electro-osmotic localization of fluorescently labeled beads rather than nonspecific protein accumulation or intrinsic fluorescence from the bead substrate. Potential thermal effects associated with electric-field application should also be considered. IgG antibodies such as those used in the present study typically exhibit thermal unfolding transitions at approximately 60–70 °C, although the precise denaturation temperature depends on antibody structure and solution conditions [31]. While local temperature was not directly measured during electrokinetic operation in the present study, a relatively low applied potential of 2 V peak-to-peak was used for bead localization. Furthermore, electrokinetic localization is required only to assemble the fluorescently labeled beads within the electrode windows; following localization, the concentrated beads remain within the detection regions after the applied field is discontinued, allowing fluorescence imaging to be performed without continued electrical operation. Consequently, prolonged application of the electric field is not required for fluorescence detection. However, direct temperature measurements would be required to quantitatively characterize potential Joule heating under the applied operating conditions.

**Figure 5:**
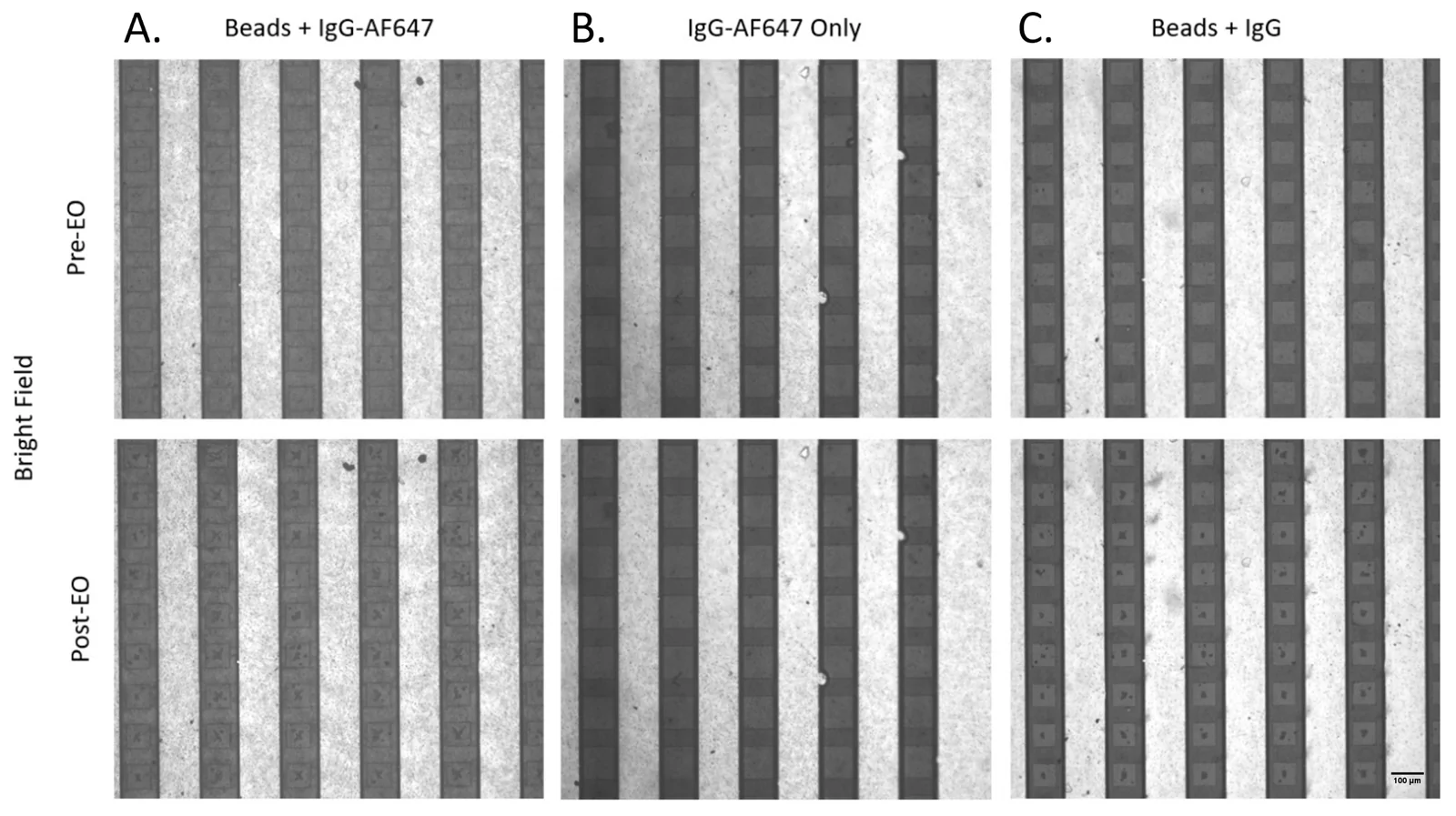
Validation of bead localization in the electrode windows using a bright-field microscope to confirm the movement of beads into the windows through electro-osmotic flow. Localization into the windows is visualized in both controls containing conjugated beads (Samples (**A**,**C**)) while absent in samples containing IgG protein only (Sample (**B**)).

Comparison of pre- and post-electro-osmosis images of samples containing fluorescently tagged protein without beads reveals that little to no assembly occurred within the windows of the electrode for non-conjugated protein in solution. This was further verified using bright field visualization of the samples with deposition of particles only visible in bead-based solutions, as Figure 5 shows, indicating that current voltage and frequency settings are appropriate for separation of unbound anti-IgG protein in solution from passively adsorbed protein on beads. Due to the ability to differentiate between beads and unbound sample in solution through guided assembly at the electrode windows, the electro-osmotic technique is a promising method for selective localization of bead-bound fluorescent signal and demonstrates the potential for selective localization of bead-bound signal in the presence of unbound fluorescent species.

### 3.2. Impact of the Protein Concentration

To determine the impact of varying protein concentrations on the fluorescent signal readout of the electro-osmotic concentrated bead, controls were tested using a range of 1 to 4 µg/mL of diluted Alexafluor-647 conjugated anti-IgG protein during incubation with the latex bead concentration of approximately 20 mg/mL. Images were taken using an exposure time of 2000 ms and a gain value of 2.

**Figure 6:**
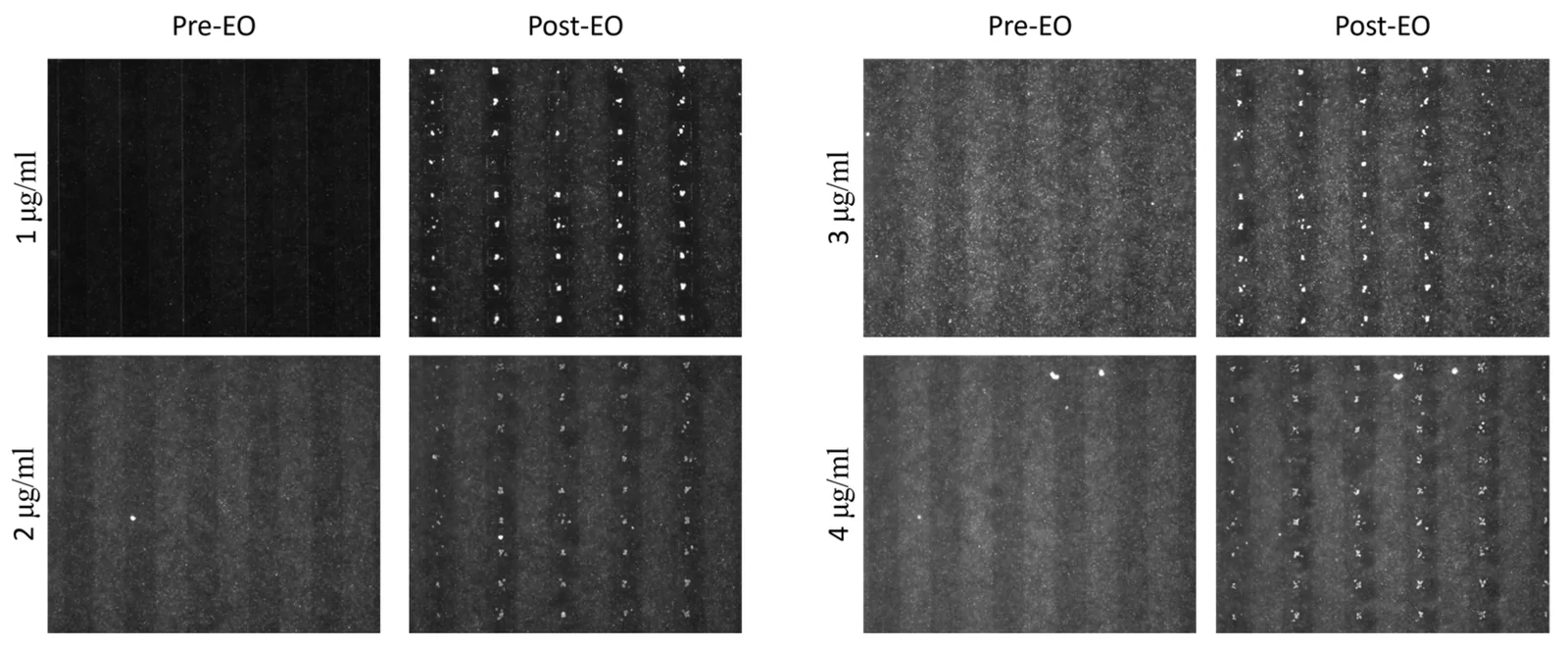
Representative conjugated polystyrene beads before (Pre-EO) and after electrokinetic preconcentration (Post-EO) at antibody fluorescence microscopy images of anti-IgG Alexa Fluor 647- concentrations of 1, 2, 3, and 4 μg/mL. Following electrokinetic manipulation, concentrated bead bands are visible along the electrode edges, demonstrating successful bead localization.

**Figure 7:**
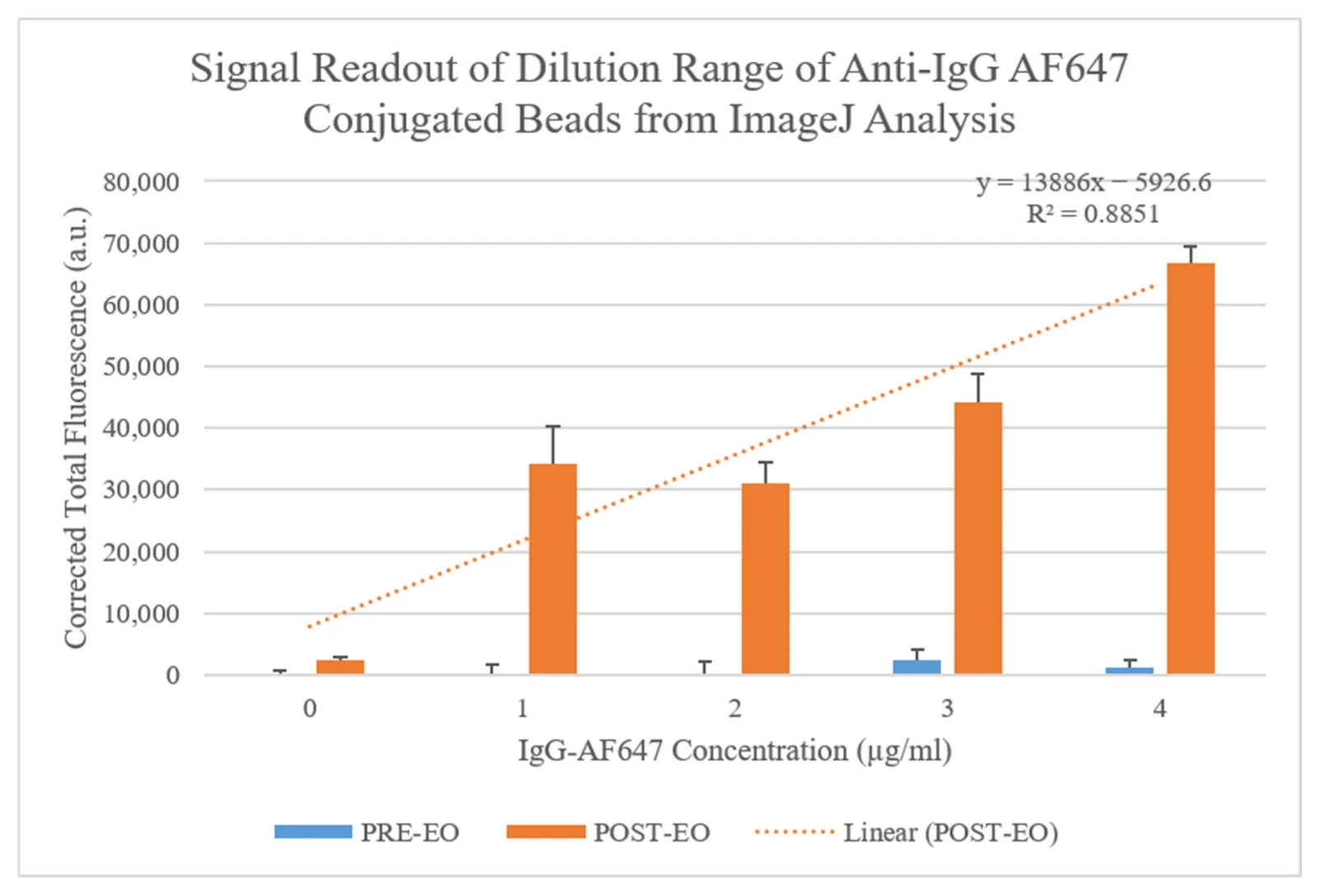
Effect of anti-IgG Alexa Fluor 647 concentration on electro-osmotic signal enhancement. Latex beads were conjugated with anti-IgG Alexa Fluor 647 at concentrations ranging from 0 to 4 μg/mL and analyzed before (Pre-EO) and after (Post-EO) electro-osmosis. Minimal fluorescence was observed prior to electro-osmosis across all concentrations, whereas post-electro-osmotic fluorescence increased substantially following bead localization within the electrode windows. Signal generally increased with increasing protein concentration, demonstrating concentration-dependent fluorescent signal enhancement following electro-osmotic concentration. Fluorescence intensity measurements were quantified using ImageJ (National Institutes of Health, Bethesda, MD, USA) with calculations performed using CTCF = Integrated Density − (Area × Mean Background). Data are presented as the mean ± standard deviation (SD) from three repeated measurements of the same window (*n* = 3). Descriptive statistical analyses were performed using Microsoft Excel (Microsoft Corporation, Redmond, WA, USA).

As the images in Figure 6 demonstrate, fluorescent signal readout was significant at all concentrations within the tested dilution range for bead passive adsorption from 1 to 4 µg/mL. To evaluate the effect of protein loading on electro-osmotic signal enhancement, latex beads were conjugated with anti-IgG Alexa Fluor 647 at concentrations ranging from 0 to 4 μg/mL and analyzed before and after electro-osmosis. Corrected total fluorescence for all five concentrations of microbeads prior and post electro-osmotic bead assembly is presented in Figure 7. Prior to electro-osmosis, corrected total fluorescence remained near background levels across all concentrations, indicating minimal localization of fluorescent signal within the electrode windows. Following electro-osmosis, a substantial increase in fluorescence was observed for all concentrations containing fluorescently labeled protein.

The IgG-AF647 concentration-dependent measurements also provide an estimate of the detection sensitivity of the method. The lowest experimentally evaluated concentration (1 µg/mL) produced a fluorescence signal of 34,304 ± 6085 a.u., substantially greater than the background signal of 2373 ± 468 a.u. Linear regression of the measurements yielded a slope of 13,886 (R^2^ = 0.885), corresponding to an estimated LOD of approximately 0.11 µg/mL using LOD=3.3 σ/S, where σ is the standard deviation of the blank and *S* is the slope of the calibration curve. Because concentrations below 1 µg/mL were not experimentally evaluated, 0.11 µg/mL should be considered an estimated rather than experimentally validated LOD. For comparison, the manufacturer recommends this anti-human IgG Fc Alexa Fluor 647 reagent at 0.5–2 µg/mL for immunocytochemistry/immunofluorescence and 1 µg/mL for flow cytometry [32]. Thus, the experimentally demonstrated detection at 1 µg/mL falls within the reagent’s recommended working range, while the estimated LOD of 0.11 µg/mL is below these typical application concentrations. Additional measurements across a broader concentration range, particularly below 1 µg/mL, would be required to experimentally validate the LOD and more fully characterize the linear range of the system.

Although the response was not strictly linear, likely due to variability in passive adsorption efficiency and washing steps, the overall trend demonstrates increased signal generation with higher levels of fluorescent protein loading on the beads. These findings indicate that electro-osmotic concentration effectively localizes fluorescently labeled beads within the electrode windows and provides substantial signal enhancement over a range of protein concentrations.

### 3.3. Spatial and Temporal Variability of Fluorescence Signal During Electro-Osmosis

Following 3 min after application of electro-osmosis to allow for bead localization to occur, samples were visualized using a Nikon Eclipse microscope (Nikon Corporation, Tokyo, Japan), and images were processed using SPOT Basic image capture software (version 5.2; SPOT Imaging, Sterling Heights, MI, USA). Fluorescent signals were measured over a period of 9 min at 3 min intervals at 10× versus 50× magnification to determine the variance of signal over time. Fluorescent signals within the windows were quantified using ImageJ analysis by subtracting the normalized signal within the window from the background signal located outside of the window.

Fluorescence intensity increased over time in all windows, indicating a consistent accumulation of signal throughout the 9 min observation period. Although the absolute fluorescence values varied between different windows, the overall trend of increasing corrected total fluorescence (CTCF) was maintained across all measured windows. Contributing factors to this window-to-window variability may include differences in the initial distribution and local concentration of fluorescently labeled beads. However, the source of the observed signal variability was not independently evaluated in this study and warrants further investigation. Despite this window-to-window variation, all windows exhibited a similar temporal increase in fluorescence, supporting a consistent signal enhancement response under the applied experimental conditions.

**Figure 8:**
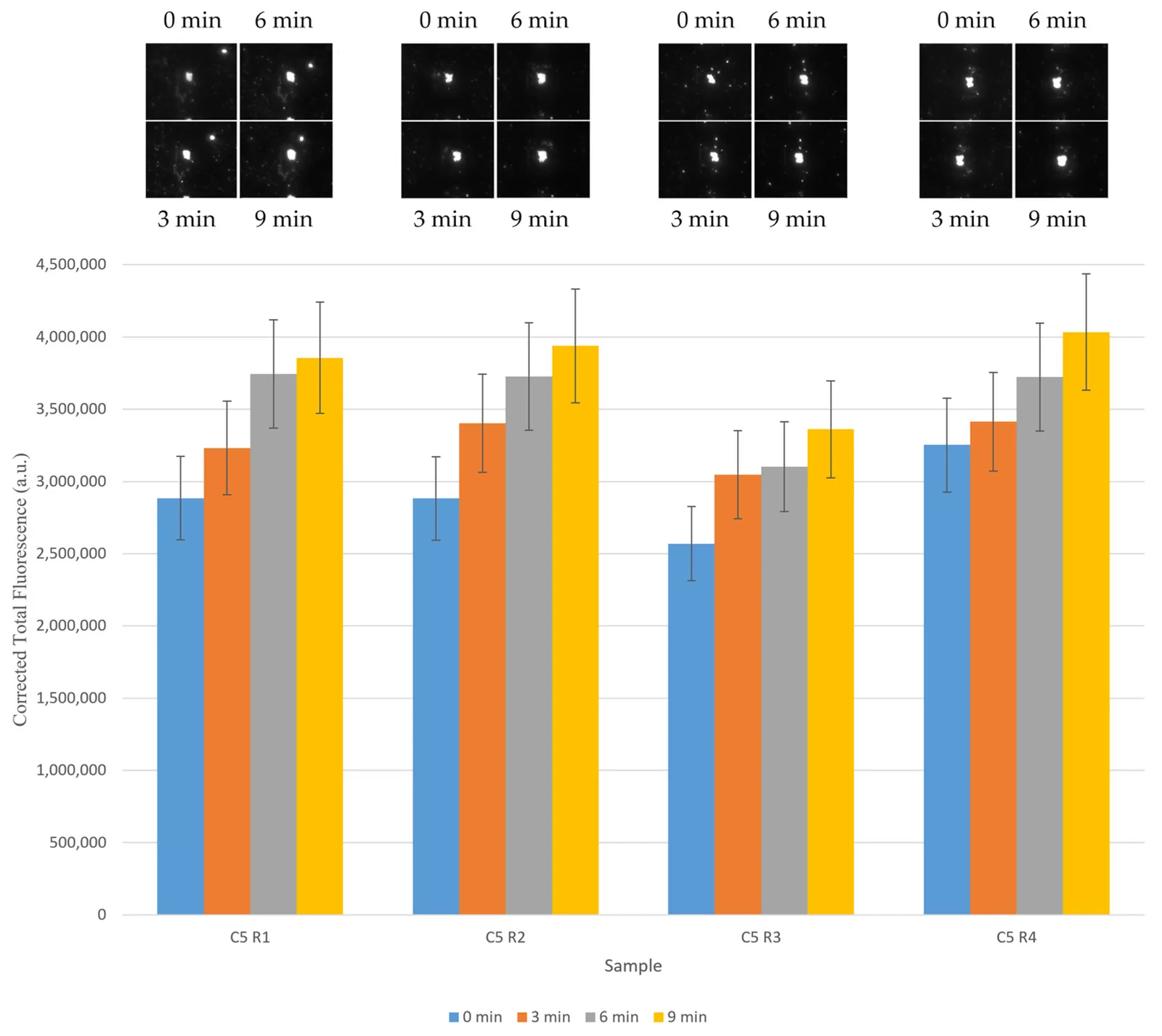
Time-dependent fluorescence signal accumulation within individual windows containing 1 μg/mL anti-IgG Alexa Fluor 647-conjugated beads during electro-osmosis. Representative fluorescence images acquired at 0, 3, 6, and 9 min are shown for four windows (C5R1–C5R4). Fluorescence intensity was quantified using ImageJ and expressed as corrected total fluorescence (CTCF) following background subtraction. All windows exhibited an increase in fluorescence signal over time, indicating progressive bead accumulation within the observation window. While the magnitude of the fluorescence signal varied between windows, the overall trend of increasing signal remained consistent across all replicates. Fluorescence intensity measurements were quantified using ImageJ (National Institutes of Health, Bethesda, MD, USA) with calculations performed using CTCF = Integrated Density − (Area × Mean Background). Data are presented as the mean ± error of 10%. Descriptive statistical analyses were performed using Microsoft Excel (Microsoft Corporation, Redmond, WA, USA).

To evaluate the temporal stability of electro-osmotic bead localization, fluorescence intensity was monitored in five individual electrode windows over a 9 min period following initiation of electro-osmosis. Corrected total fluorescence was measured at time intervals of 0, 3, 6, and 9 min using ImageJ analysis. All four windows exhibited progressive increases in fluorescence over time as demonstrated in Figure 8, indicating continued accumulation of fluorescently labeled beads within the electrode windows. The largest increases generally occurred during the first 3–6 min, after which signal growth began to plateau, suggesting that bead localization approaches equilibrium within the observed time frame. Although absolute fluorescence intensity varied between windows, the temporal trends were consistent across all five locations.

The results demonstrating a high rate of fluorescent signal increase within the first 3 to 6 min support the attractive possibility of achieving short sample-to-answer times in biological assays.

**Figure 9:**
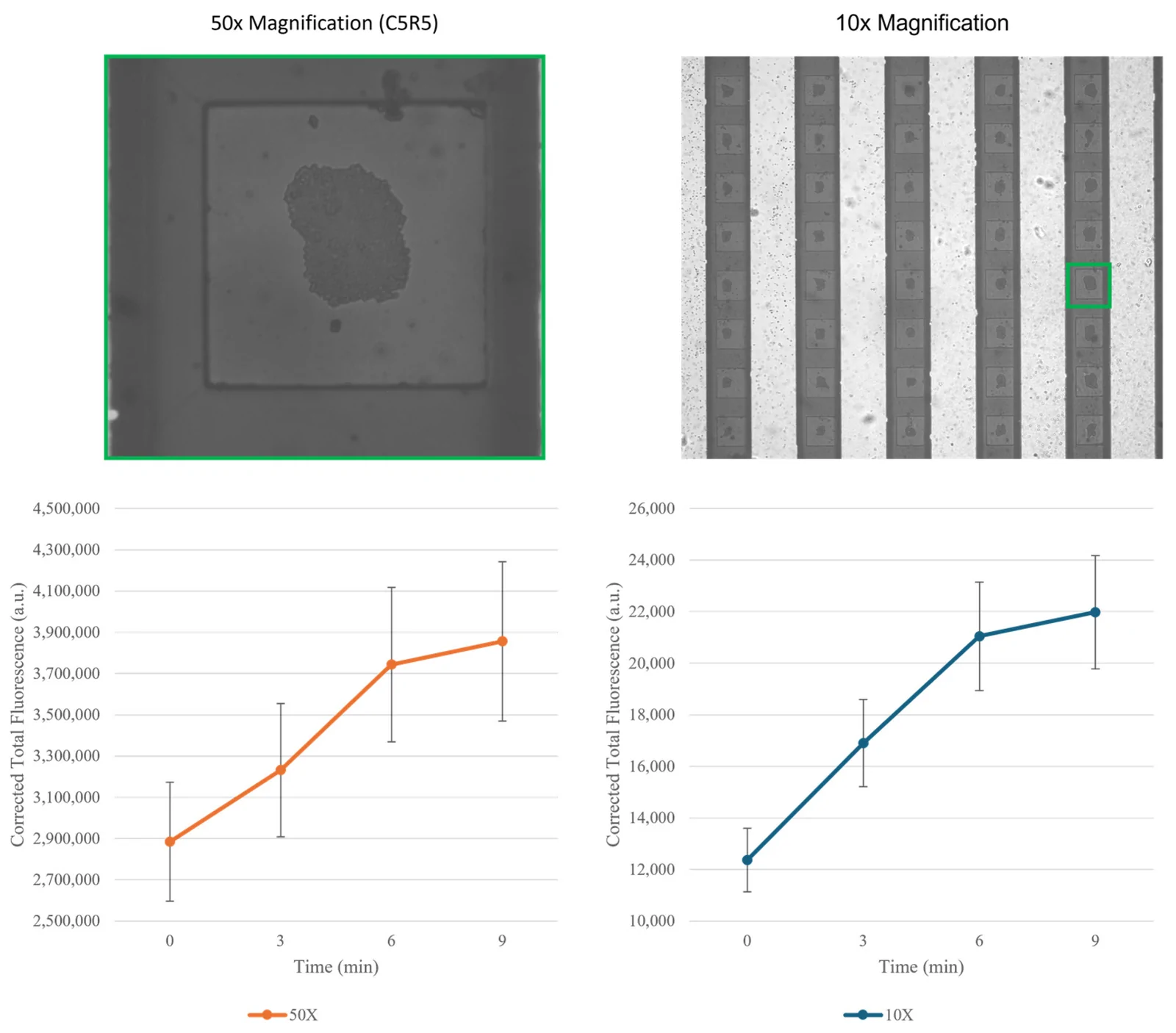
Effect of microscope magnification on fluorescence signal quantification during electro-osmotic bead accumulation. Corrected total fluorescence (CTCF) was measured from images acquired at 10× and 50× magnification at time intervals of 0, 3, 6, and 9 min during electro-osmosis of 1 μg/mL anti-IgG Alexa Fluor 647-conjugated beads.

The effect of microscope magnification on fluorescence signal quantification during electro-osmotic bead accumulation was studied for 10× and 50× magnification of the Nikon Eclipse microscope. Corrected total fluorescence (CTCF) was measured from images acquired at 10× and 50× magnification at time intervals of 0, 3, 6, and 9 min during electro-osmosis of 1 μg/mL anti-IgG Alexa Fluor 647-conjugated beads. Representative fields of view for each magnification are presented in Figure 9. Fluorescence intensity increased over time at both magnifications, indicating progressive bead accumulation within the observation window. Higher CTCF values were observed at 50× magnification due to the increased spatial resolution and improved visualization of bead aggregates. Fluorescence intensity measurements were quantified using ImageJ (National Institutes of Health, Bethesda, MD, USA) with calculations performed using CTCF = Integrated Density − (Area × Mean Background). Data are presented as the mean ± error of 10%. Descriptive statistical analyses were performed using Microsoft Excel (Microsoft Corporation, Redmond, WA, USA).

### 3.4. Electro-Osmotic Concentration of TNF-α Conjugated Beads

To evaluate the applicability of electro-osmotic bead concentration to a biologically relevant assay, a TNF-α sandwich immunoassay was adapted to the latex bead platform and analyzed before and after electro-osmosis. The human tumor necrosis factor alpha (TNF-α) is the prototypic ligand of the TNF superfamily and plays a central role in inflammation, immune system development, apoptosis, and lipid metabolism. TNF has been found to induce cell death in certain tumor cell lines but can also play an oncogenic role and serve as an important biomarker for inflammation-induced cancer [33,34,35]. Electro-osmotic concentration was further tested using the TNF-α Human Matched Antibody Pair from Thermo Fisher Scientific (Thermo Fisher Scientific, Waltham, MA, USA) due to its relevance in biomarker detection and an established protocol for implementation on the latex bead-based assay (see Appendix A).

**Figure 10:**
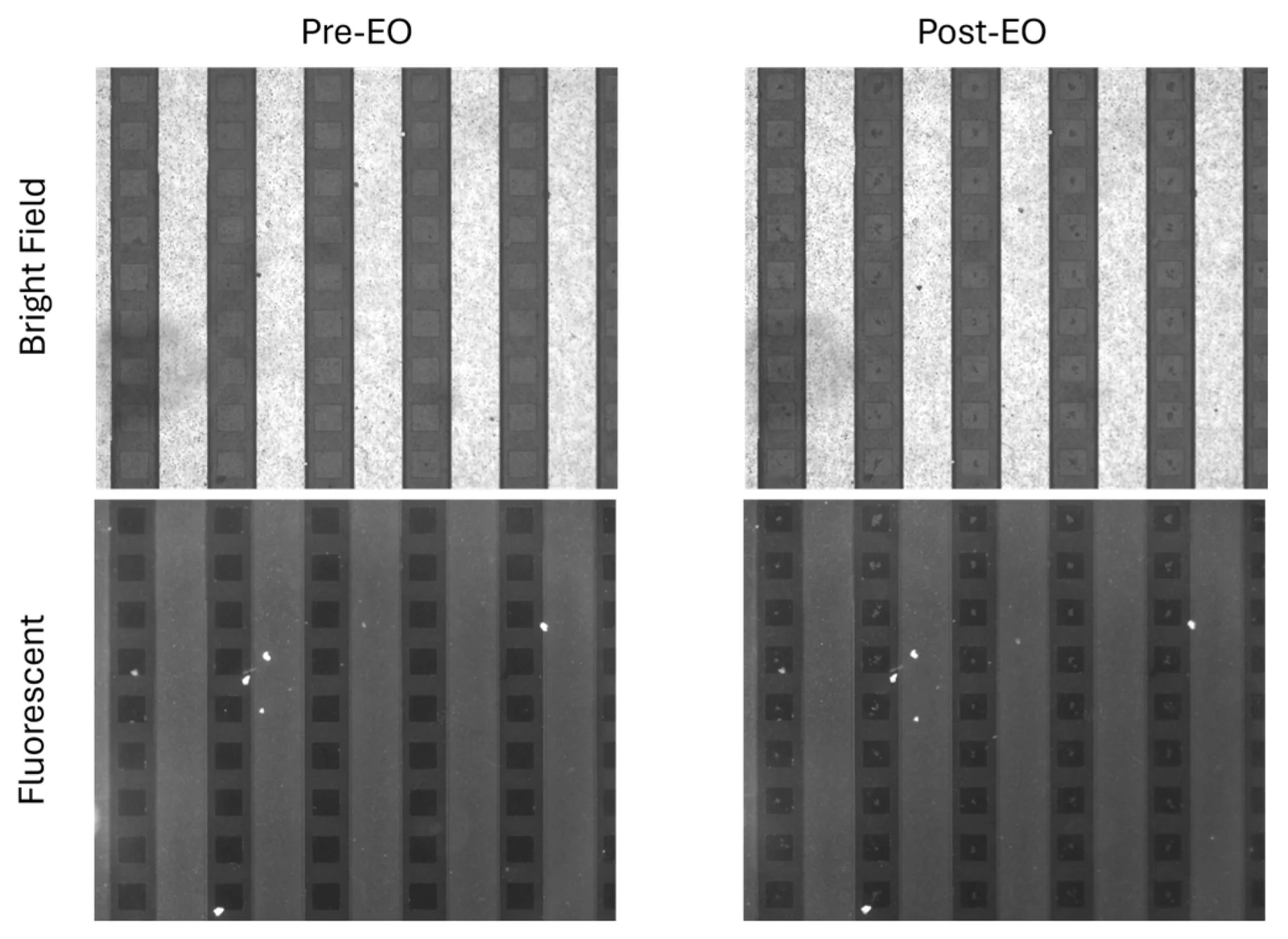
Representative bright-field (**top**) and fluorescence (**bottom**) microscopy images of TNF-α-conjugated beads before (Pre-EO) and after (Post-EO) electro-osmotic concentration. Bright-field images illustrate the window orientation and bead migration, while fluorescence images reveal enhanced fluorescence signal after electro-osmosis, consistent with the accumulation and concentration of TNF-α-conjugated beads within the device.

**Figure 11:**
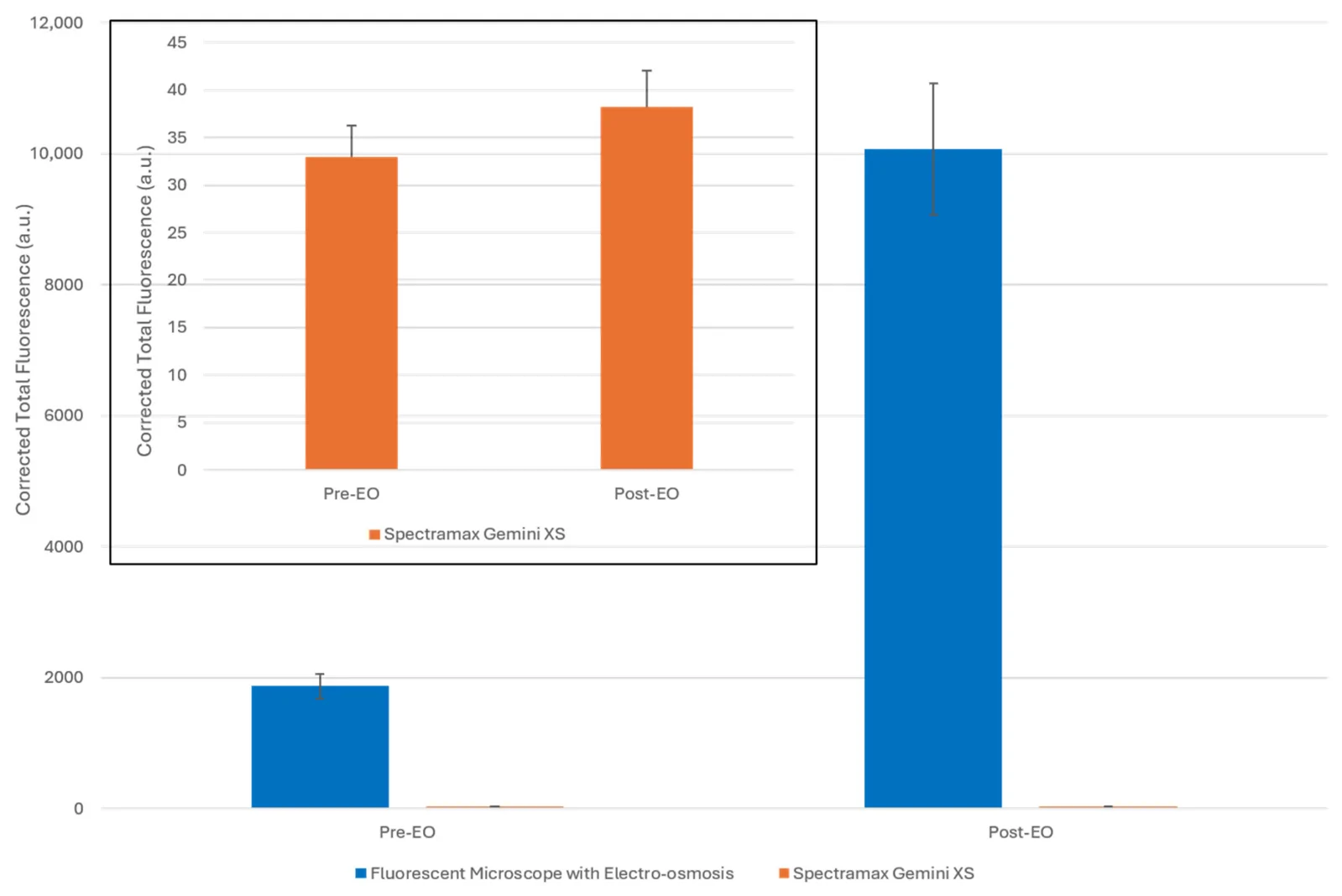
Comparison of TNF-α-conjugated bead fluorescence quantified by ImageJ analysis of fluorescence microscopy images and a SPECTRAmax GEMINI XS plate reader before and after electro-osmosis. Fluorescence microscopy yielded significantly higher corrected fluorescence values and a substantially larger dynamic range than the conventional plate reader, allowing the EO-induced increase in signal to be clearly resolved. Conversely, fluorescence measured with the plate reader remained near background levels, demonstrating that microscopy-based detection more effectively resolves the localized EO-induced fluorescence increase under the conditions evaluated. Fluorescence intensity measurements were quantified using ImageJ to measure corrected total fluorescence with calculations performed using CTCF = Integrated Density − (Area × Mean Background). Data are presented as the mean ± error of 10%. Descriptive statistical analyses were performed using Microsoft Excel.

Prior to electro-osmotic (EO) manipulation, the assay produced a corrected fluorescence signal of approximately 1800 a.u. Following EO, fluorescence increased to approximately 10,000 a.u., corresponding to an approximately 5.5-fold enhancement in signal intensity. This increase is attributed to the localization of fluorescent bead conjugates into discrete regions within the electrode windows, resulting in a substantially higher local fluorescence intensity than that observed in the dispersed bead suspension as shown in Figure 10. Under the same experimental conditions, fluorescence measurements obtained from the bulk suspension using the SPECTRAmax^®^ GEMINI XS plate reader remained near background levels as the localized signal enhancement generated by EO was not captured by bulk fluorescence measurements. Therefore, imaging-based fluorescence detection provides a more appropriate approach for quantifying the degree of signal enhancement achieved through EO-mediated bead localization than bulk fluorescence measurement techniques, such as microplate readers. TNF-α conjugated beads demonstrated a significant increase in corrected total fluorescence following electro-osmotic bead assembly, as data presented in Figure 11 indicate.

The TNF-α experiment in the present study was performed at a single concentration of 2000 pg/mL as a proof-of-concept demonstration of electrokinetic post-assay signal enhancement. Accordingly, these results demonstrate enhancement of the localized fluorescence signal following electrokinetic manipulation but do not establish the analytical performance of the TNF-α assay. Another important distinction to be made in the present study is that control experiments were designed to evaluate electrokinetic localization and sources of fluorescence background rather than immunological selectivity for TNF-α. Although free fluorescent protein exhibited minimal electrokinetic localization and HRP-conjugated beads demonstrated negligible intrinsic fluorescence, these controls do not distinguish fluorescence resulting from specific TNF-α binding from fluorescence arising through nonspecific adsorption of assay reagents to the bead surface. Therefore, the present TNF-α experiment demonstrates the compatibility of electrokinetic post-assay signal enhancement with a bead-based sandwich immunoassay but does not independently establish the immunological selectivity of the TNF-α assay. Selectivity in the presence of potentially interfering proteins was not evaluated in the present study and will require validation in future applications of this work. Electrokinetic bead localization may also be influenced by physicochemical properties of the suspending medium, including electrical conductivity, ionic strength, pH, viscosity, and protein content. In the present study, sample preparation conditions were standardized through consistent washing and blocking procedures, followed by resuspension of the beads in 1× PBS and a 1:10 dilution in UltraPure distilled water immediately prior to electrokinetic testing. However, the electrical conductivity and other physicochemical properties of the final suspensions were not directly measured. Therefore, potential variability in residual upstream assay components following the washing procedures and their effects on electrokinetic bead localization cannot be independently determined from the present data. Characterization of these parameters will be important for further optimization and standardization of the platform across different assay conditions.

TNF-α detection has been demonstrated using a range of biosensing strategies with varying signal enhancement mechanisms and analytical sensitivities. For example, a microfluidic photonic crystal enhanced fluorescence immunoassay reported a TNF-α limit of detection (LOD) of 1.57 pg/mL, while a fluorescent silica nanoparticle-based immunoassay achieved an LOD of 0.1 pg/mL [36,37]. Electrochemical approaches have similarly demonstrated high analytical sensitivity, including an impedance-based magnetic bead sensor with an LOD of 1 pg/mL in undiluted human serum and a DNA aptamer-based electrochemical sensor with reported LODs of 0.07 pg/mL in buffer and 0.14 pg/mL in diluted human serum [38,39]. In contrast to these approaches, the present method utilizes electrokinetic localization of fluorescent bead-bound complexes as a post-assay signal enhancement mechanism without requiring enzymatic amplification or modification of the underlying fluorescence detection chemistry. However, because the present study was limited to a proof-of-concept demonstration of electrokinetic signal enhancement and was not used to establish a TNF-α calibration curve, limit of detection, or dynamic range, direct quantitative comparisons of analytical performance with previously reported TNF-α biosensors cannot be made.

## 4. Conclusions

The presented study demonstrated post-assay fluorescent signal enhancement for bead-based immunoassays provided by electro-osmotic (EO) particle localization. By concentrating analyte-bound fluorescent beads within defined electrode windows, EO increases local fluorescence intensity relative to signal distributed throughout the bulk solution, providing an additional stage of signal enhancement without requiring enzymatic amplification or additional labeling chemistries. The preferential localization of bead-bound complexes, while free fluorescent protein exhibited minimal accumulation, further suggests that the electrokinetic process possesses inherent size-selective enrichment capabilities. This behavior may improve assay specificity by preferentially enriching bead-bound signal over smaller unbound fluorescent species that contribute to background fluorescence, thereby enhancing signal-to-background ratios. In addition, because EO produces highly localized regions of fluorescence, imaging-based detection methods, such as fluorescence microscopy, are particularly well suited for quantifying EO-induced signal enhancement by measuring fluorescence within these confined regions rather than averaging signal across the entire sample volume. This localized detection strategy may also enable analysis using smaller sample volumes than conventional bulk fluorescence approaches, offering the potential to reduce sample and reagent consumption in future assay implementations.

Beyond its analytical advantages, EO provides an integrated strategy for immobilization-based assays by enabling the self-assembly of bead-bound analytes directly within the detection region. While the present study retained conventional washing steps prior to electrokinetic localization, the ability to selectively localize bead-bound signal may support future development of simplified assay workflows with reduced separation, resuspension, or transfer requirements. By integrating analyte localization and fluorescence detection within a single platform, EO has the potential to simplify assay workflows while remaining compatible with scalable microfluidic devices. Although additional optimization and validation across a broader range of biomarkers and assay formats are warranted, these findings demonstrate the potential of EO-mediated bead localization as a versatile post-assay signal enhancement strategy for fluorescence-based laboratory assays and future low-cost point-of-care diagnostic applications.

## Data Availability

The original contributions presented in this study are included in the article/Supplementary Material. Further inquiries can be directed to the corresponding author.

## Supplementary Materials

The following supporting information can be downloaded at: https://www.mdpi.com/article/10.3390/s26165264/s1, File S1: Passive adsorption of protein to carboxyl-modified latex beads.

## Appendix A. TNF-α ELISA Protocol Adaptation for Latex Beads

Pre-titrated, purified anti-Human TNF-α coating antibody was prepared at a concentration of 0.08 µg/mL in 0.025 M MES buffer and incubated at a 1:1 ratio with 500 μL of previously prepared 1 μm carboxyl-modified latex (CML) polystyrene beads (Thermo Fisher Scientific) at 25 °C overnight for 10 h and centrifuged at 3000 rpm for 20 min. Supernatant was then removed and resuspended with 1 mL 1× PBS (Thermo Fisher Scientific) and centrifuged again to sediment the particles. This wash step was repeated for a total of four washes and then blocked in 250 μL 1× StartingBlock™ (PBS) blocking buffer (Thermo Fisher Scientific) for 2 h at 25 °C. Samples were then centrifuged again to sediment the particles at 3000 rpm for 20 min and washed in 400 μL 1× PBS. Wash steps were performed for a total of four washes. Recombinant human TNF-α standard was prepared at a concentration of 2000 pg/mL in 100 μL of 1× assay buffer (8.0 g NaCl, 1.13 g Na_2_HPO_4_, 0.2 g KH_2_PO_4_, 0.2 g KCl, 5.0 g bovine serum albumin (fraction V), 1 mL Tween 20, 0.5% ProClinTM in 1 mL DI H_2_O) and incubated with 100 μL of the coating antibody-conjugated latex beads at 25 °C for 60 min. Samples were then centrifuged to sediment the particles at 3000 rpm for 20 min and washed in 400 μL 1× PBS. Wash steps were performed for a total of four washes. Biotinylated anti-Human TNF-α detection antibodies were prepared at a concentration of 1:200 in 1× assay buffer to a volume of 200 μL and incubated with the latex beads at 25 °C for 60 min. Samples were then centrifuged again to sediment the particles at 3000 rpm for 20 min and washed in 400 μL 1× PBS. Wash steps were performed for a total of four washes. Streptavidin-modified Alexa Fluor™ 647 conjugate (Thermo Fisher Scientific) was prepared to a dilution of 0.05 mg/mL in 100 μL and incubated with the latex beads for 40 min at 25 °C. Samples were then centrifuged to sediment the particles at 3000 rpm for 20 min and washed in 400 μL 1× PBS. Wash steps were performed for a total of four washes. Beads were then resuspended in 500 μL 1× PBS and placed in storage. Prior to electro-osmosis testing on the electrodes, bead samples were immediately prepared at a 1:10 dilution in UltraPure™ DNase/RNase-Free Distilled Water (Thermo Fisher Scientific).
