# Supplementary material for: Guided Electrokinetic Assembly of Functionalized Latex Beads for Fluorescence Signal Enhancement Using AC Electro-Osmosis
Source: Sensors (Basel). 2026 Aug 20;26(16):5264. doi: 10.3390/s26165264 (PMC13517280; doi:10.3390/s26165264)
Supplement: Supplementary file 1 [file sensors-26-05264-s001.zip › sensors-4462192-supplementary.pdf]

<u>Supplementary Materials File S1</u>

Passive adsorption of protein to carboxyl-modified latex beads [30]

Latex preparation

Carboxyl-modified latex microspheres (ThermoFisher Scientific, Waltham, MA, USA) at concentrations of 40mg/mL were prepared by adding 2.5 mL of the bead suspension to 10 mL of 0.025 M MES buffer (ThermoFisher Scientific, Waltham, MA, USA). The suspension was centrifuged at 3,000 × g for 20 min, after which the supernatant was discarded and the pellet was resuspended in 10 mL of fresh MES buffer. The washing step was repeated by centrifugation under the same conditions, followed by removal of the supernatant. The final pellet was resuspended in 5 mL of 0.025 M MES buffer, yielding a latex suspension with an approximate concentration of 20 mg/mL (2% solids).

Passive adsorption

The protein or antibody was prepared at a concentration of 1 mg/mL in 0.025 M MES buffer. Five milliliters of the latex suspension (20 mg/mL) was combined with 5 mL of the associated Goat anti-Human IgG protein solution (ThermoFisher Scientific, Waltham, MA, USA) and incubated overnight at room temperature with gentle mixing to allow passive adsorption onto the bead surface. Following incubation, the suspension was centrifuged to pellet the protein-coated latex particles, and the supernatant was removed. The pellet was resuspended in 10 mL of PBS (0.1 M, pH 7.2), centrifuged, and washed twice more with PBS for a total of three washes. The final pellet was resuspended in 10 mL of storage buffer (PBS containing 0.1% glycine and 0.1% sodium azide), resulting in an approximate bead concentration of 1% solids. The coated particles were stored at 4 °C until use.
